# Crystal structure of *N*-[3-(benzo[*d*]thia­zol-2-yl)-6-bromo-2*H*-chromen-2-yl­idene]-4-methyl­benzenamine

**DOI:** 10.1107/S2056989023002979

**Published:** 2023-04-14

**Authors:** Amira E. M. Abdallah, Galal H. Elgemeie, Peter G. Jones

**Affiliations:** a Chemistry Department, Faculty of Science, Helwan University, Cairo, Egypt; b Institut für Anorganische und Analytische Chemie, Technische Universität Braunschweig, Hagenring 30, D-38106 Braunschweig, Germany; Universität Greifswald, Germany

**Keywords:** crystal structure, benzo[*d*]thia­zole, chromene, imine, π–π-stacking

## Abstract

The title compound, C_23_H_15_BrN_2_OS, was the unexpected product in an attempted synthesis of the isomeric 3-(benzo[*d*]thia­zol-2-yl)-6-bromo-1-*p*-tolyl­quinolin-2(1*H*)-one. The C_chromene_=N—C angle is wide [125.28 (8)°]. The benzo­thia­zole and chromene ring systems are almost coplanar, with their planes parallel to (1  0); the toluene ring system is rotated by *ca* 40° out of the chromene plane. The mol­ecular packing involves layers with π-stacking, borderline ‘weak’ hydrogen bonds and possible C—H⋯π contacts.

## Abstract 2

In the crystal structure of the title compound, the C=N—C angle is wide [125.28 (8)°]. The benzo­thia­zole and chromene ring systems are almost coplanar and lie parallel to (1 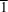 0); the toluene ring system is rotated by *ca* 40° out of the chromene plane.

## 1. Chemical context

**Figure 1:**
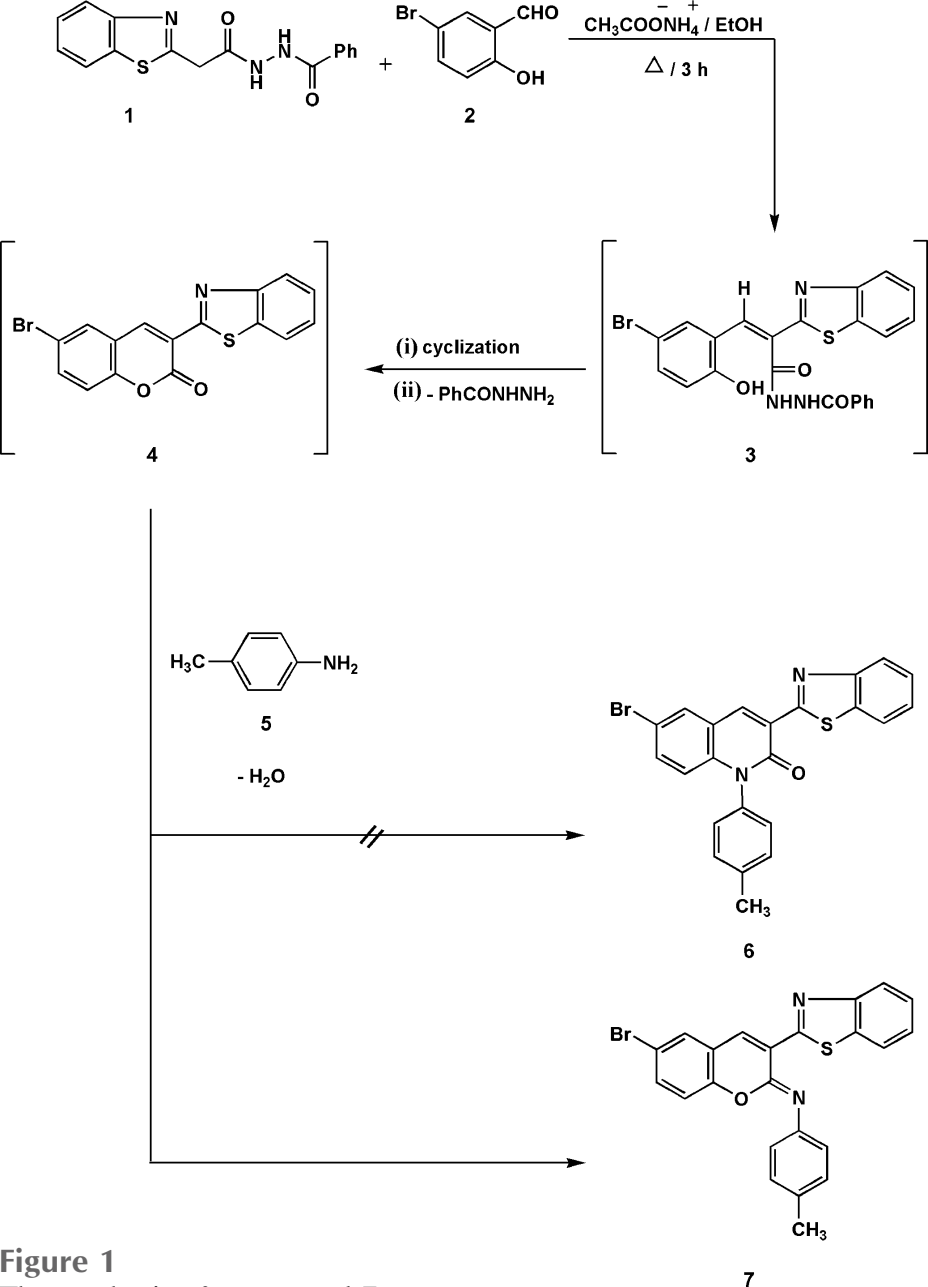
The synthesis of compound **7**.

Benzo­thia­zoles exhibit strong fluorescence and luminescence properties (Wang *et al.*, 2010 ▸). Incorporated benzo­thia­zole moieties are present in many commercially important organofluorescent materials that have attracted significant research inter­est in the field of organic light-emitting diodes (Lu *et al.*, 2017 ▸; Metwally *et al.*, 2022*a* ▸,*b* ▸). Coumarin (IUPAC name 2*H*-chromen-2-one) is a natural product and flavouring agent. Recently, a series of novel benzo­thia­zolyl-coumarin hybrids have been synthesized as potential biological agents and efficient emitting materials (Azzam *et al.*, 2021 ▸, 2022*a* ▸,*b* ▸,*c* ▸,*d* ▸; Wu *et al.*, 2011 ▸). We have previously prepared 3-(benzo[*d*]oxazol, -imidazole, -thia­zol-2-yl)-2*H*-chromen-2-imine and their corresponding coumarin analogues 3-(benzo[*d*]oxazol-, -imidazol, -thia­zol-2-yl)-2*H*-chromen-2-one, through the reaction of salicyl­aldehyde with 2-cyano­methyl-benzoxazole, -benzimidazole, and -benzo­thia­zole, respectively (Elgemeie, 1989 ▸). Some derivatives of these ring systems, known commercially as coumarin-6, coumarin-7 and coumarin-30, have been used as laser dyes in medical applications (Das *et al.*, 2021 ▸; Satpati *et al.*, 2009 ▸). Recently, we have synthesized some coumarin derivatives that exhibit fluorescence properties (Elgemeie & Elghandour, 1990 ▸; Elgemeie *et al.*, 2000*a* ▸,*b* ▸; Elgemeie *et al.*, 2015 ▸) as part of our research inter­est in exploiting new coumarin and benzo­thia­zole deriv­atives for biological and photochemical materials (Azzam *et al.*, 2017*a* ▸,*b* ▸, 2020*a* ▸,*b* ▸,*c* ▸,*d* ▸; Metwally *et al.*, 2021*a* ▸,*b* ▸). Here, we describe a one-pot reaction of *N*-[2-(benzo[*d*]thia­zol-2-yl)acet­yl]benzohydrazide (**1**) with 5-bromo-salicylaldehde (**2**) and 4-*p*-toluidine (**5**) (Fig. 1 ▸). The mass spectrum of the product was, however, inconsistent with the proposed structure, 3-(benzo[*d*]thia­zol-2-yl)-6-bromo-1-*p*-tolyl­quinolin-2(1*H*)-one (**6**). Therefore, the X-ray crystal structure was determined, showing the exclusive presence of *N*-[3-(benzo[*d*]thiazol-2-yl)-6-bromo-2*H*-chromen-2-yl­idene]-4-methyl­benz­en­a­mine (**7**), an isomer of **6**, as the sole product in the solid state; this was unexpected because the C=O moiety of the coumarin framework is usually chemically robust. The formation of **7** presumably involves the initial formation of the adduct **3** followed by elimination of benzohydrazide; the inter­mediate **4** then reacts with *p*-toluidine to give the final product **7** by elimination of water. 
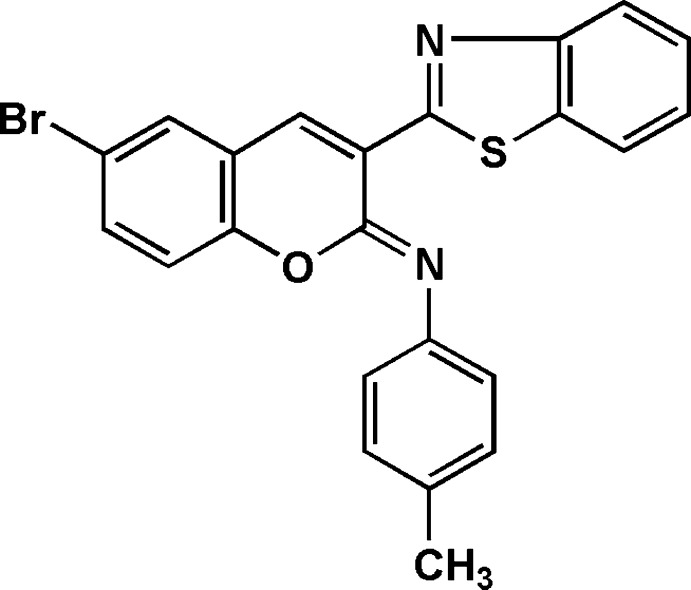


## 2. Structural commentary

**Figure 2:**
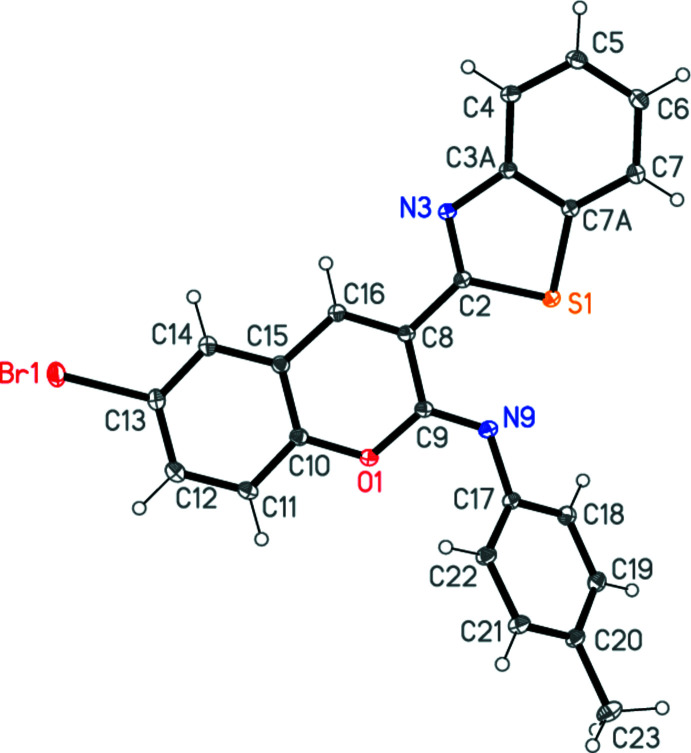
The mol­ecule of compound **7** in the crystal. Ellipsoids represent 50% probability levels.

The mol­ecule of **7** is shown in Fig. 2 ▸. The structure determ­ination makes clear that the unexpected product is a chromene derivative with an exocyclic imino function rather than a quinoline with an exocyclic oxo function. Bond lengths and angles may be regarded as normal, except that the C9=N9—C17 angle is very wide at 125.28 (8)°; selected values are given in Table 1 ▸. The benzo­thia­zole and chromene ring systems are almost coplanar, with an inter­planar angle of 7.59 (2)°; associated with this is a short intra­molecular contact S1⋯N9 2.7570 (8) Å. The toluene ring system is appreciably rotated out of the chromene plane, with an inter­planar angle of 40.38 (2)°.

## 3. Supra­molecular features

**Figure 3:**
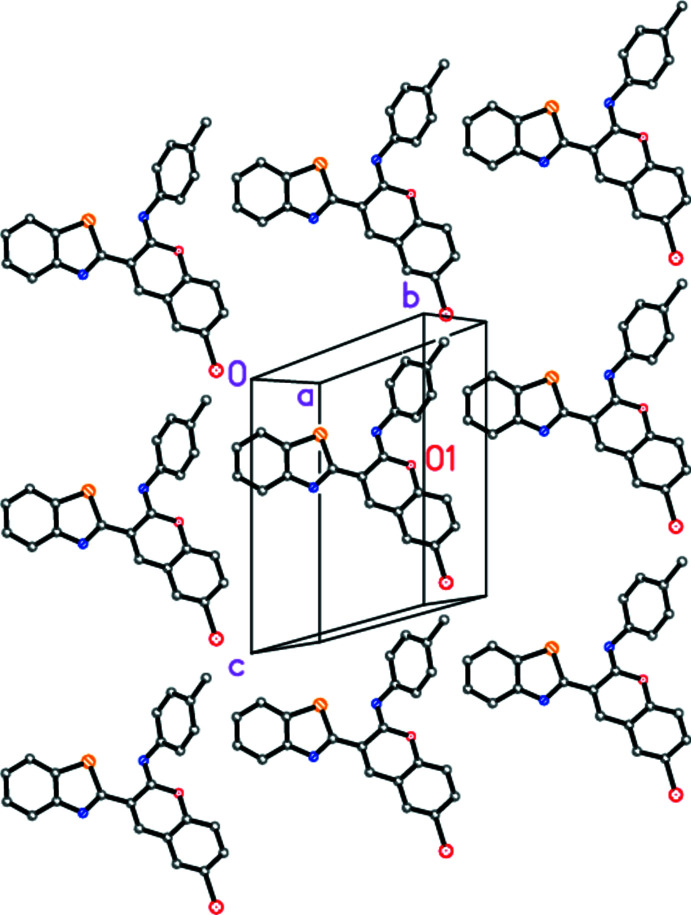
Layer structure of compound **7** (without hydrogen atoms) showing the asymmetric unit (indicated by the label O1) and further translation-related mol­ecules viewed perpendicular to the plane (1 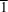 0). A second layer is related to the first by inversion.

**Figure 4:**
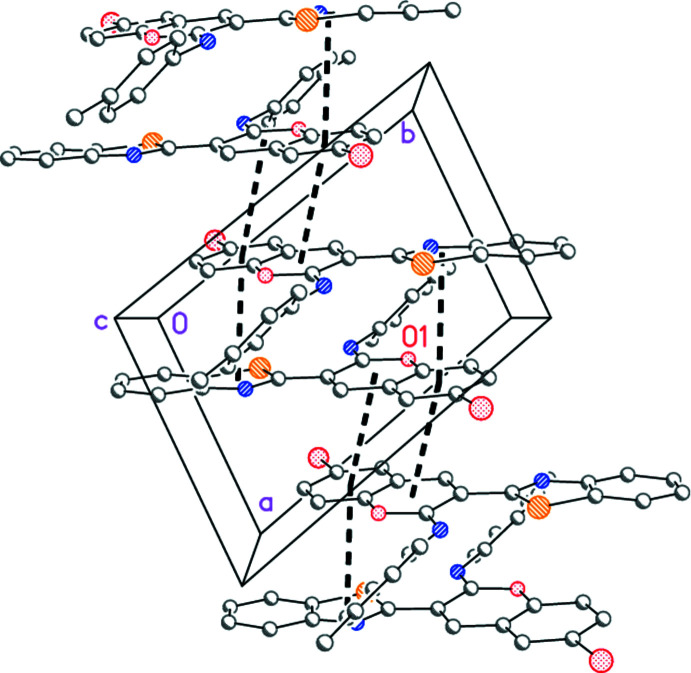
Stacking of ring systems in the structure of **7** (without hydrogen atoms). The view direction is parallel to the *c* axis. The label O1 indicates the mol­ecule of the chosen asymmetric unit.

There are few short contacts between mol­ecules; two borderline ‘weak’ hydrogen bonds are listed in Table 2 ▸. A tenable packing analysis attributes a central role to the ring systems; individual rings are denoted here as *A* (thia­zole), *B* (benzo ring of benzo­thia­zole), *C* (pyran ring of chromene), *D* (benzo ring of chromene) and *E* (tol­yl). The mol­ecules lie with rings *A*–*D* almost parallel to (1 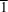 0) (Fig. 3 ▸), and there are weak stacking effects *A*⋯*D* [inter­centroid distance 3.5910 (5) Å, offset 1.12 Å; operator 1 − *x*, 1 − *y*, 1 − *z*], *C*⋯*C* [3.6184 (5) Å, 1.35 Å; 2 − *x*, 1 − *y*, 1 − *z*] and *C*⋯*D* [3.6308 (5) Å, 1.27 Å; 2 − *x*, 1 − *y*, 1 − *z*] (Fig. 4 ▸). Two possible C—H⋯π inter­actions are represented by the contacts H21⋯*Cg*(*B*) [*Cg* = centroid; H⋯*Cg* 2.89 Å, C—H⋯*Cg* 122°; −1 + *x*, −1 + *y*, *z*] and H6⋯*Cg*(*E*) [H⋯*Cg* 2.87 Å, C—H⋯*Cg* 124°; *x*, 1 + *y*, *z*]; the angles are narrow, but the inter­actions do not necessarily involve the ring centroids. The contacts H7⋯Br1 and H6⋯*Cg*(*E*) lie within the parent layer; H22⋯N3 is formed to a neighbouring layer and H21⋯*Cg*(*B*) to the next layer but one.

## 4. Database survey

The searches employed the routine ConQuest (Bruno *et al.*, 2002 ▸), part of Version 2022.3.0 of the CSD (Groom *et al.*, 2016 ▸).

We recently reported the structure of the mixed coumarin/benzo[*d*]thia­zole derivative 3-(benzo[*d*]thia­zol-2-yl)-2*H*-chromen-2-one [3-(1,3-benzo­thia­zol-2-yl)-2*H*-1-benzo­pyran-2-one] (Abdallah *et al.*, 2022 ▸). The structure of the 4-oxo isomer had already been published by Lohar *et al.* (2018 ▸). Two more related structures were published by others at the same time (Singh *et al.*, 2022 ▸). The current structure, however, bears an imine (=NAr) rather than an oxo substituent at atom C2 of the chromene (and thus is strictly not a coumarin). Only one other such structure was found in the database; its substituent at the imine nitro­gen atom is pyridin-2-ethyl (refcode ITEVAF; Ahamed & Ghosh, 2011 ▸) and its C=N—C angle is much narrower than in **7** at 118.5 (7)°. A further search was therefore performed for structures with an =NAr group at the 2-position of a chromene ring system. This gave 18 hits with a considerable spread of C=N—C angles, namely 120.5–127.9°, mean value 123.4 (24)°. Nine of these structures appeared in the same publication (Shishkina *et al.*, 2019 ▸), and, like **7**, none of them had an inter­planar angle close to the calculated gas-phase optimum of 0°.

## 5. Synthesis and crystallization

5-Bromo-salicyl­aldehyde **2** (2.01 g, 0.01 mol), *p*-toluidine **5** (1.07 g, 0.01 mol) and solid ammonium acetate (0.77 g, 0.01 mol) were added to a solution of *N*-[2-(benzo[*d*]thia­zol-2-yl)acet­yl]benzohydrazide **1** (3.11 g, 0.01 mol) in ethanol (25 mL). The reaction mixture was refluxed for 3 h, and the solid thus formed was collected by filtration and recrystallized from ethanol.

Yellow crystals (seen under the microscope to be orange/yellow dichroic); yield: 94% (4.21 g); m.p. 501–503 K; IR (KBr, cm^−1^): ν 3052, (CH-aromatic), 2918, 2852 (CH_3_), 1554 (C=N), 1591, 1476 (C=C). ^1^H NMR (400 MHz, DMSO-*d*_6_) *δ*: 2.51 (*s*, 3H, CH_3_), 7.16–8.28 (*m*, 11H, 2 C_6_H_4_, C_6_H_3_), 8.73 (*s*, 1H, CH-pyran). ^13^C NMR (100 MHz, DMSO-*d_6_*) *δ*: 21.2 (CH_3_), 116.5, 118.0, 121.7, 122.5 (2), 123.1, 123.9, 125.8, 127.0, 129.9 (2), 132.0, 134.1, 134.5, 135.1, 137.6, 141.7, 145.6, 152.0, 152.1 (aromatic carbons, pyran ring), 160.5 (C=N). Analysis: calculated for C_23_H_15_BrN_2_OS (447.35): C 61.75, H 3.38, N 6.26, S 7.17%. Found: C 61.86, H 3.50, N 6.06, S 6.99%.

## 6. Refinement

Crystal data, data collection and structure refinement details are summarized in Table 3 ▸. The methyl group was included as an idealized rigid group allowed to rotate but not tip (C—H 0.98 Å, H—C—H 109.5°). Other hydrogen atoms were included using a riding model starting from calculated positions, with C—H 0.95 Å. The *U*(H) values were fixed at 1.5 × *U*_eq_ of the parent carbon atoms for methyl H atoms and 1.2 × *U*_eq_ for other hydrogen atoms.

## Supplementary Material

Crystal structure: contains datablock(s) I, global. DOI: 10.1107/S2056989023002979/yz2032sup1.cif

Structure factors: contains datablock(s) I. DOI: 10.1107/S2056989023002979/yz2032Isup2.hkl

Supporting information file. DOI: 10.1107/S2056989023002979/yz2032Isup3.cml

CCDC reference: 2252955

Additional supporting information:  crystallographic information; 3D view; checkCIF report

## Figures and Tables

**Table 1:** Selected geometric parameters (Å, °)

S1—C7*A*	1.7340 (9)	C9—O1	1.3819 (11)
S1—C2	1.7512 (9)	O1—C10	1.3751 (12)
C2—N3	1.3094 (12)	N9—C17	1.4127 (12)
C2—C8	1.4705 (12)	C13—Br1	1.8969 (10)
C9—N9	1.2708 (12)		
			
C7*A*—S1—C2	88.85 (4)	C3*A*—C7*A*—S1	109.69 (7)
N3—C2—S1	115.73 (7)	C10—O1—C9	121.82 (7)
C2—N3—C3*A*	110.78 (8)	C9—N9—C17	125.28 (8)
N3—C3*A*—C7*A*	114.92 (8)		

**Table 2:** Hydrogen-bond geometry (Å, °)

*D*—H⋯*A*	*D*—H	H⋯*A*	*D*⋯*A*	*D*—H⋯*A*
C7—H7⋯Br1^i^	0.95	3.11	3.7721 (10)	128
C22—H22⋯N3^ii^	0.95	2.63	3.5716 (13)	169

Symmetry codes: (i) 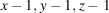 ; (ii) 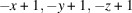 .

**Table 3:** Experimental details

Crystal data	
Chemical formula	C_23_H_15_BrN_2_OS
*M*_r_	447.34
Crystal system, space group	Triclinic, *P* 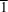
Temperature (K)	100
*a*, *b*, *c* (Å)	7.34138 (10), 10.6720 (2), 12.9247 (2)
α, β, γ (°)	104.5034 (16), 90.2462 (12), 103.9961 (14)
*V* (Å^3^)	948.97 (3)
*Z*	2
Radiation type	Mo *K*α
μ (mm^−1^)	2.29
Crystal size (mm)	0.20 × 0.15 × 0.12

Data collection	
Diffractometer	XtaLAB Synergy
Absorption correction	Multi-scan (*CrysAlis PRO*; Rigaku OD, 2022 ▸)
*T*_min_, *T*_max_	0.902, 1.000
No. of measured, independent and observed [*I* > 2σ(*I*)] reflections	126966, 12477, 11717
*R*_int_	0.030
(sin θ/λ)_max_ (Å^−1^)	0.928

Refinement	
*R*[*F* ^2^ > 2σ(*F* ^2^)], *wR*(*F* ^2^), *S*	0.044, 0.086, 1.27
No. of reflections	12477
No. of parameters	254
H-atom treatment	H-atom parameters constrained
Δρ_max_, Δρ_min_ (e Å^−3^)	1.00, −0.64

Computer programs: *CrysAlis PRO* (Rigaku OD, 2022 ▸), *SHELXT* (Sheldrick, 2015*b* ▸), *SHELXL2018/3* (Sheldrick, 2015*a* ▸) and *XP* (Siemens, 1994 ▸).

## supplementary crystallographic information

### Crystal data

**Table:**

C_23_H_15_BrN_2_OS	*Z* = 2
*M**_r_* = 447.34	*F*(000) = 452
Triclinic, *P*1	*D*_x_ = 1.566 Mg m^−^^3^
*a* = 7.34138 (10) Å	Mo *K*α radiation, λ = 0.71073 Å
*b* = 10.6720 (2) Å	Cell parameters from 55045 reflections
*c* = 12.9247 (2) Å	θ = 2.3–41.0°
α = 104.5034 (16)°	µ = 2.29 mm^−^^1^
β = 90.2462 (12)°	*T* = 100 K
γ = 103.9961 (14)°	Block, yellow-orange dichroic
*V* = 948.97 (3) Å^3^	0.20 × 0.15 × 0.12 mm

### Data collection

**Table:**

XtaLAB Synergy diffractometer	12477 independent reflections
Radiation source: micro-focus sealed X-ray tube, PhotonJet (Mo) X-ray Source	11717 reflections with *I* > 2σ(*I*)
Mirror monochromator	*R*_int_ = 0.030
Detector resolution: 10.0000 pixels mm^-1^	θ_max_ = 41.3°, θ_min_ = 2.0°
ω scans	*h* = −13→13
Absorption correction: multi-scan (CrysAlisPro; Rigaku OD, 2022)	*k* = −19→19
*T*_min_ = 0.902, *T*_max_ = 1.000	*l* = −23→23
126966 measured reflections	

### Refinement

**Table:**

Refinement on *F*^2^	Primary atom site location: dual
Least-squares matrix: full	Hydrogen site location: inferred from neighbouring sites
*R*[*F*^2^ > 2σ(*F*^2^)] = 0.044	H-atom parameters constrained
*wR*(*F*^2^) = 0.086	*w* = 1/[σ^2^(*F*_o_^2^) + (0.0329*P*)^2^ + 0.3775*P*] where *P* = (*F*_o_^2^ + 2*F*_c_^2^)/3
*S* = 1.27	(Δ/σ)_max_ = 0.003
12477 reflections	Δρ_max_ = 1.00 e Å^−^^3^
254 parameters	Δρ_min_ = −0.64 e Å^−^^3^
0 restraints	

### Special details

**Table:**

Geometry. All esds (except the esd in the dihedral angle between two l.s. planes) are estimated using the full covariance matrix. The cell esds are taken into account individually in the estimation of esds in distances, angles and torsion angles; correlations between esds in cell parameters are only used when they are defined by crystal symmetry. An approximate (isotropic) treatment of cell esds is used for estimating esds involving l.s. planes. Short intramolecular contact: 2.7570 (0.0008) S1 - N9 Least-squares planes (x,y,z in crystal coordinates) and deviations from them (* indicates atom used to define plane) 6.9114 (0.0010) x + 0.6551 (0.0043) y + 0.9221 (0.0052) z = 5.0880 (0.0023) * 0.0005 (0.0007) C17 * -0.0080 (0.0007) C18 * 0.0078 (0.0007) C19 * -0.0001 (0.0007) C20 * -0.0074 (0.0007) C21 * 0.0071 (0.0007) C22 -0.1433 (0.0014) N9 0.0078 (0.0018) C23 Rms deviation of fitted atoms = 0.0062 6.7905 (0.0005) x - 4.3737 (0.0026) y - 3.5046 (0.0028) z = 1.1567 (0.0016) Angle to previous plane (with approximate esd) = 40.378 ( 0.018 ) * -0.0112 (0.0007) C8 * -0.0356 (0.0007) C9 * 0.0189 (0.0007) O1 * 0.0164 (0.0008) C10 * 0.0078 (0.0008) C11 * -0.0097 (0.0008) C12 * -0.0229 (0.0008) C13 * -0.0003 (0.0008) C14 * 0.0198 (0.0008) C15 * 0.0168 (0.0007) C16 -0.1250 (0.0009) Br1 -0.0945 (0.0011) N9 Rms deviation of fitted atoms = 0.0184 6.6853 (0.0007) x - 5.5698 (0.0018) y - 2.4072 (0.0037) z = 1.1618 (0.0013) Angle to previous plane (with approximate esd) = 7.587 ( 0.024 ) * -0.0246 (0.0005) S1 * -0.0299 (0.0006) C2 * 0.0114 (0.0007) N3 * 0.0329 (0.0008) C3A * 0.0037 (0.0008) C4 * -0.0325 (0.0008) C5 * -0.0172 (0.0009) C6 * 0.0192 (0.0008) C7 * 0.0370 (0.0008) C7A Rms deviation of fitted atoms = 0.0254

### Fractional atomic coordinates and isotropic or equivalent isotropic displacement parameters (Å^2^)

**Table:**

	*x*	*y*	*z*	*U*_iso_*/*U*_eq_	
S1	0.45005 (3)	0.22816 (2)	0.24952 (2)	0.01102 (4)	
C2	0.53265 (12)	0.27006 (9)	0.38418 (7)	0.00988 (12)	
N3	0.47875 (11)	0.17627 (8)	0.43436 (6)	0.01092 (11)	
C3A	0.36335 (12)	0.06293 (9)	0.36717 (7)	0.01059 (12)	
C4	0.27384 (14)	−0.05271 (10)	0.39829 (8)	0.01396 (14)	
H4	0.294443	−0.059470	0.469083	0.017*	
C5	0.15488 (14)	−0.15658 (10)	0.32326 (9)	0.01606 (16)	
H5	0.091595	−0.234805	0.343368	0.019*	
C6	0.12611 (14)	−0.14821 (10)	0.21763 (9)	0.01639 (16)	
H6	0.044937	−0.221294	0.167457	0.020*	
C7	0.21439 (14)	−0.03497 (10)	0.18570 (8)	0.01469 (15)	
H7	0.195092	−0.029543	0.114386	0.018*	
C7A	0.33289 (12)	0.07121 (9)	0.26171 (7)	0.01109 (13)	
C8	0.65444 (12)	0.40075 (9)	0.44104 (7)	0.01000 (12)	
C9	0.69589 (12)	0.51144 (9)	0.39019 (7)	0.01044 (12)	
O1	0.81113 (10)	0.63197 (7)	0.44747 (6)	0.01236 (11)	
N9	0.62892 (12)	0.49654 (8)	0.29581 (7)	0.01224 (12)	
C10	0.87706 (12)	0.65155 (9)	0.55151 (7)	0.01079 (12)	
C11	0.98434 (13)	0.77861 (9)	0.60325 (8)	0.01284 (14)	
H11	1.010923	0.847551	0.567020	0.015*	
C12	1.05226 (13)	0.80324 (10)	0.70912 (8)	0.01385 (14)	
H12	1.126245	0.889327	0.745864	0.017*	
C13	1.01084 (13)	0.70054 (10)	0.76081 (8)	0.01306 (14)	
Br1	1.09469 (2)	0.73707 (2)	0.90683 (2)	0.01723 (3)	
C14	0.90564 (13)	0.57339 (9)	0.70921 (8)	0.01269 (13)	
H14	0.880313	0.504533	0.745531	0.015*	
C15	0.83692 (12)	0.54769 (9)	0.60237 (7)	0.01077 (12)	
C16	0.72340 (12)	0.41962 (9)	0.54312 (7)	0.01106 (13)	
H16	0.696241	0.347267	0.575708	0.013*	
C17	0.64703 (13)	0.59939 (9)	0.24293 (7)	0.01134 (13)	
C18	0.66395 (14)	0.56395 (10)	0.13211 (8)	0.01423 (14)	
H18	0.674436	0.476506	0.097566	0.017*	
C19	0.66550 (15)	0.65591 (10)	0.07226 (8)	0.01490 (15)	
H19	0.680212	0.631073	−0.002518	0.018*	
C20	0.64577 (13)	0.78423 (10)	0.12042 (8)	0.01351 (14)	
C21	0.62680 (14)	0.81793 (10)	0.23071 (8)	0.01380 (14)	
H21	0.611872	0.904390	0.264656	0.017*	
C22	0.62919 (13)	0.72820 (9)	0.29233 (8)	0.01265 (13)	
H22	0.618755	0.754253	0.367493	0.015*	
C23	0.64635 (18)	0.88259 (12)	0.05472 (10)	0.02078 (19)	
H23A	0.613539	0.961990	0.098977	0.031*	
H23B	0.554014	0.841125	−0.006738	0.031*	
H23C	0.771864	0.908668	0.029148	0.031*	

### Atomic displacement parameters (Å^2^)

**Table:**

	*U*^11^	*U*^22^	*U*^33^	*U*^12^	*U*^13^	*U*^23^
S1	0.01261 (8)	0.01125 (8)	0.00922 (8)	0.00140 (6)	0.00060 (6)	0.00424 (6)
C2	0.0102 (3)	0.0100 (3)	0.0097 (3)	0.0025 (2)	0.0010 (2)	0.0031 (2)
N3	0.0119 (3)	0.0103 (3)	0.0107 (3)	0.0012 (2)	0.0001 (2)	0.0044 (2)
C3A	0.0103 (3)	0.0104 (3)	0.0113 (3)	0.0019 (2)	0.0004 (2)	0.0040 (2)
C4	0.0142 (3)	0.0127 (3)	0.0152 (4)	0.0007 (3)	−0.0004 (3)	0.0067 (3)
C5	0.0144 (3)	0.0132 (3)	0.0200 (4)	−0.0004 (3)	−0.0013 (3)	0.0070 (3)
C6	0.0151 (4)	0.0131 (3)	0.0185 (4)	−0.0005 (3)	−0.0031 (3)	0.0036 (3)
C7	0.0155 (3)	0.0136 (3)	0.0131 (4)	0.0007 (3)	−0.0021 (3)	0.0029 (3)
C7A	0.0113 (3)	0.0111 (3)	0.0107 (3)	0.0021 (2)	0.0004 (2)	0.0034 (2)
C8	0.0101 (3)	0.0096 (3)	0.0107 (3)	0.0023 (2)	0.0011 (2)	0.0036 (2)
C9	0.0104 (3)	0.0096 (3)	0.0116 (3)	0.0018 (2)	0.0014 (2)	0.0038 (2)
O1	0.0136 (3)	0.0105 (2)	0.0122 (3)	0.0001 (2)	−0.0009 (2)	0.0043 (2)
N9	0.0145 (3)	0.0111 (3)	0.0116 (3)	0.0022 (2)	0.0009 (2)	0.0049 (2)
C10	0.0100 (3)	0.0104 (3)	0.0120 (3)	0.0023 (2)	0.0009 (2)	0.0032 (2)
C11	0.0122 (3)	0.0100 (3)	0.0154 (4)	0.0011 (2)	0.0009 (3)	0.0033 (3)
C12	0.0127 (3)	0.0117 (3)	0.0155 (4)	0.0020 (3)	0.0002 (3)	0.0015 (3)
C13	0.0126 (3)	0.0138 (3)	0.0116 (3)	0.0029 (3)	−0.0006 (3)	0.0016 (3)
Br1	0.01926 (5)	0.01788 (5)	0.01143 (4)	0.00218 (3)	−0.00160 (3)	0.00057 (3)
C14	0.0132 (3)	0.0125 (3)	0.0119 (3)	0.0027 (3)	−0.0004 (3)	0.0028 (3)
C15	0.0101 (3)	0.0103 (3)	0.0118 (3)	0.0023 (2)	0.0004 (2)	0.0029 (2)
C16	0.0113 (3)	0.0104 (3)	0.0116 (3)	0.0022 (2)	0.0004 (2)	0.0036 (2)
C17	0.0124 (3)	0.0111 (3)	0.0106 (3)	0.0015 (2)	0.0002 (2)	0.0043 (2)
C18	0.0184 (4)	0.0125 (3)	0.0108 (3)	0.0021 (3)	0.0001 (3)	0.0029 (3)
C19	0.0184 (4)	0.0156 (4)	0.0099 (3)	0.0017 (3)	0.0000 (3)	0.0043 (3)
C20	0.0142 (3)	0.0147 (3)	0.0127 (4)	0.0020 (3)	0.0002 (3)	0.0068 (3)
C21	0.0161 (3)	0.0131 (3)	0.0140 (4)	0.0046 (3)	0.0025 (3)	0.0060 (3)
C22	0.0153 (3)	0.0127 (3)	0.0112 (3)	0.0040 (3)	0.0025 (3)	0.0048 (3)
C23	0.0267 (5)	0.0210 (4)	0.0186 (5)	0.0057 (4)	0.0018 (4)	0.0125 (4)

### Geometric parameters (Å, º)

**Table:**

S1—C7A	1.7340 (9)	C15—C16	1.4382 (13)
S1—C2	1.7512 (9)	C17—C18	1.4010 (13)
C2—N3	1.3094 (12)	C17—C22	1.4010 (13)
C2—C8	1.4705 (12)	C18—C19	1.3915 (14)
N3—C3A	1.3809 (12)	C19—C20	1.3977 (15)
C3A—C4	1.4055 (13)	C20—C21	1.3964 (14)
C3A—C7A	1.4082 (13)	C20—C23	1.5057 (14)
C4—C5	1.3849 (14)	C21—C22	1.3934 (13)
C5—C6	1.4085 (15)	C4—H4	0.9500
C6—C7	1.3872 (14)	C5—H5	0.9500
C7—C7A	1.4015 (13)	C6—H6	0.9500
C8—C16	1.3600 (13)	C7—H7	0.9500
C8—C9	1.4616 (12)	C11—H11	0.9500
C9—N9	1.2708 (12)	C12—H12	0.9500
C9—O1	1.3819 (11)	C14—H14	0.9500
O1—C10	1.3751 (12)	C16—H16	0.9500
N9—C17	1.4127 (12)	C18—H18	0.9500
C10—C11	1.3896 (13)	C19—H19	0.9500
C10—C15	1.3985 (13)	C21—H21	0.9500
C11—C12	1.3931 (14)	C22—H22	0.9500
C12—C13	1.3954 (14)	C23—H23A	0.9800
C13—C14	1.3849 (13)	C23—H23B	0.9800
C13—Br1	1.8969 (10)	C23—H23C	0.9800
C14—C15	1.4049 (13)		
			
C7A—S1—C2	88.85 (4)	C22—C17—N9	123.92 (8)
N3—C2—C8	120.14 (8)	C19—C18—C17	120.48 (9)
N3—C2—S1	115.73 (7)	C18—C19—C20	121.05 (9)
C8—C2—S1	124.12 (6)	C21—C20—C19	117.95 (9)
C2—N3—C3A	110.78 (8)	C21—C20—C23	121.46 (9)
N3—C3A—C4	124.80 (8)	C19—C20—C23	120.58 (9)
N3—C3A—C7A	114.92 (8)	C22—C21—C20	121.80 (9)
C4—C3A—C7A	120.23 (8)	C21—C22—C17	119.69 (9)
C5—C4—C3A	118.40 (9)	C5—C4—H4	120.8
C4—C5—C6	121.12 (9)	C3A—C4—H4	120.8
C7—C6—C5	121.06 (9)	C4—C5—H5	119.4
C6—C7—C7A	118.06 (9)	C6—C5—H5	119.4
C7—C7A—C3A	121.11 (8)	C7—C6—H6	119.5
C7—C7A—S1	129.11 (7)	C5—C6—H6	119.5
C3A—C7A—S1	109.69 (7)	C6—C7—H7	121.0
C16—C8—C9	119.94 (8)	C7A—C7—H7	121.0
C16—C8—C2	119.48 (8)	C10—C11—H11	120.5
C9—C8—C2	120.55 (8)	C12—C11—H11	120.5
N9—C9—O1	121.20 (8)	C11—C12—H12	120.2
N9—C9—C8	120.80 (8)	C13—C12—H12	120.2
O1—C9—C8	118.00 (8)	C13—C14—H14	120.5
C10—O1—C9	121.82 (7)	C15—C14—H14	120.5
C9—N9—C17	125.28 (8)	C8—C16—H16	119.6
O1—C10—C11	117.00 (8)	C15—C16—H16	119.6
O1—C10—C15	121.11 (8)	C19—C18—H18	119.8
C11—C10—C15	121.89 (9)	C17—C18—H18	119.8
C10—C11—C12	118.90 (9)	C18—C19—H19	119.5
C11—C12—C13	119.52 (9)	C20—C19—H19	119.5
C14—C13—C12	121.77 (9)	C22—C21—H21	119.1
C14—C13—Br1	119.00 (7)	C20—C21—H21	119.1
C12—C13—Br1	119.21 (7)	C21—C22—H22	120.2
C13—C14—C15	119.05 (9)	C17—C22—H22	120.2
C10—C15—C14	118.86 (8)	C20—C23—H23A	109.5
C10—C15—C16	118.19 (8)	C20—C23—H23B	109.5
C14—C15—C16	122.95 (8)	H23A—C23—H23B	109.5
C8—C16—C15	120.85 (8)	C20—C23—H23C	109.5
C18—C17—C22	119.00 (8)	H23A—C23—H23C	109.5
C18—C17—N9	116.72 (8)	H23B—C23—H23C	109.5
			
C7A—S1—C2—N3	0.05 (7)	C9—O1—C10—C11	−176.93 (8)
C7A—S1—C2—C8	−179.77 (8)	C9—O1—C10—C15	2.96 (13)
C8—C2—N3—C3A	179.03 (8)	O1—C10—C11—C12	179.31 (8)
S1—C2—N3—C3A	−0.79 (10)	C15—C10—C11—C12	−0.57 (14)
C2—N3—C3A—C4	−176.34 (9)	C10—C11—C12—C13	−0.22 (14)
C2—N3—C3A—C7A	1.36 (11)	C11—C12—C13—C14	0.92 (15)
N3—C3A—C4—C5	177.03 (9)	C11—C12—C13—Br1	−177.29 (7)
C7A—C3A—C4—C5	−0.56 (14)	C12—C13—C14—C15	−0.80 (14)
C3A—C4—C5—C6	1.09 (16)	Br1—C13—C14—C15	177.41 (7)
C4—C5—C6—C7	−0.75 (17)	O1—C10—C15—C14	−179.19 (8)
C5—C6—C7—C7A	−0.15 (16)	C11—C10—C15—C14	0.68 (13)
C6—C7—C7A—C3A	0.68 (15)	O1—C10—C15—C16	−0.42 (13)
C6—C7—C7A—S1	−175.47 (8)	C11—C10—C15—C16	179.46 (8)
N3—C3A—C7A—C7	−178.14 (9)	C13—C14—C15—C10	0.00 (13)
C4—C3A—C7A—C7	−0.33 (14)	C13—C14—C15—C16	−178.71 (9)
N3—C3A—C7A—S1	−1.32 (10)	C9—C8—C16—C15	0.14 (13)
C4—C3A—C7A—S1	176.50 (7)	C2—C8—C16—C15	−177.59 (8)
C2—S1—C7A—C7	177.19 (10)	C10—C15—C16—C8	−1.08 (13)
C2—S1—C7A—C3A	0.69 (7)	C14—C15—C16—C8	177.64 (9)
N3—C2—C8—C16	5.52 (13)	C9—N9—C17—C18	145.41 (10)
S1—C2—C8—C16	−174.66 (7)	C9—N9—C17—C22	−41.55 (14)
N3—C2—C8—C9	−172.19 (8)	C22—C17—C18—C19	0.84 (14)
S1—C2—C8—C9	7.62 (12)	N9—C17—C18—C19	174.24 (9)
C16—C8—C9—N9	−178.29 (9)	C17—C18—C19—C20	−1.56 (15)
C2—C8—C9—N9	−0.59 (13)	C18—C19—C20—C21	0.79 (15)
C16—C8—C9—O1	2.26 (13)	C18—C19—C20—C23	−179.58 (10)
C2—C8—C9—O1	179.97 (8)	C19—C20—C21—C22	0.68 (15)
N9—C9—O1—C10	176.74 (8)	C23—C20—C21—C22	−178.95 (10)
C8—C9—O1—C10	−3.82 (12)	C20—C21—C22—C17	−1.37 (15)
O1—C9—N9—C17	−6.20 (14)	C18—C17—C22—C21	0.59 (14)
C8—C9—N9—C17	174.37 (8)	N9—C17—C22—C21	−172.29 (9)

### Hydrogen-bond geometry (Å, º)

**Table:**

*D*—H···*A*	*D*—H	H···*A*	*D*···*A*	*D*—H···*A*
C7—H7···Br1^i^	0.95	3.11	3.7721 (10)	128
C22—H22···N3^ii^	0.95	2.63	3.5716 (13)	169

Symmetry codes: (i) *x*−1, *y*−1, *z*−1; (ii) −*x*+1, −*y*+1, −*z*+1.
